# The immunostimulatory effects and pro‐apoptotic activity of rhCNB against Lewis lung cancer is mediated by Toll‐like receptor 4

**DOI:** 10.1002/cam4.2158

**Published:** 2019-06-20

**Authors:** Jinju Yang, Hongwei Zhang, Ziwei Zhu, Yadan Gao, Benqiong Xiang, Qun Wei

**Affiliations:** ^1^ Department of Biochemistry and Molecular Biology, Gene Engineering and Biotechnology Beijing Key Laboratory Beijing Normal University Beijing P. R. China; ^2^ National Key Laboratory of Biochemical Engineering, Institute of Process Engineering Chinese Academy of Sciences Beijing China

**Keywords:** antitumor drug, antitumor immunity, innate immunity, rhCNB, Toll‐like receptor 4

## Abstract

**Background:**

Recombinant human calcineurin B subunit (rhCNB) has been shown to be an immune‐stimulatory protein promoting cytokine production and inducing phenotypic maturation of Dendritic cells (DCs). In vivo, it has good antitumor efficacy, and has potential as an antitumor drug. Exogenous rhCNB was found to be internalized into tumor cells via the Toll‐like receptor 4 (TLR4) complex, but it was not known whether its immuno‐modulatory and antitumor functions involved entry by this same route.

**Methods:**

The production and secretion of the cytokines and chemokines in innate immune cells induced by rhCNB were determined by ELISA, and the expression of CD40, CD80, CD86, and MHCII was analyzed by FACs. Experimental Lewis lung cancer (LLC) model was prepared in C57 BL/6 wild‐type (WT) mice, TLR4^−/−^ mice or their littermates by the inoculation of LLCs in their right armpit, and then administrated daily intraperitoneal injections (0.2 mL) of normal saline, rhCNB 20 mg/kg, and rhCNB 40 mg/kg, respectively.

**Results:**

Recombinant human calcineurin B subunit promoted the production of antitumor cytokines by innate immune cells, and culture supernatants of rhCNB‐stimulated immune cells induced apoptosis of LLCs. In addition, rhCNB up‐regulated CD40, CD80, CD86, and MHCII expression in macrophages and DCs in TLR4^+^ cells but failed to do so in TLR4 deficient cells. rhCNB also induced the formation of CD4^+^ and CD8^+^T cells in splenocytes from WT mice, but not from TLR4‐deficient littermates. Intraperitoneal administration of WT C57BL/6 mice with rhCNB resulted in a 50% reduction in LLC tumor growth, but failed to inhibit tumor growth in TLR4^−/−^ littermates.

**Conclusions:**

The in vivo antitumor and immunomodulatory effects of rhCNB are mediated by the TLR4. This conclusion is important for the further understanding and development of rhCNB as an antitumor drug.

## 1 INTRODUCTION

The calcineurin B subunit (CNB) is the regulatory subunit of calcineurin (CN). CN is composed of A and B subunits, and plays a pivotal role in activating immune cells and regulating apoptosis via both transcriptional and posttranscriptional routes. The basic function of CNB is to control the function of the catalytic subunit, subunit A,^1^, ^2^, ^3^, ^4^ but it has other activities. It is involved in the proteasome pathway via its interactions with heat shock protein 60, tubulin, procaspase 3 and other proteins^5^ and deficiency for CNB leads to a high risk of squamous cell carcinoma.^6^ Our previous research showed that recombinant human CNB (rhCNB) had good antitumor efficacy against a variety of tumor models: it inhibited tumor growth in the H22 xenograft model, promoted tumor regression in the S180 sarcoma xenograft model, and had significant antitumor activity in HepG2, SK‐HEP‐1 xenograft models and the B16 melanoma metastasis model.^7^, ^8^ Also recent data from the Safety Review Center indicate that rhCNB suppresses tumor progression in a variety of tumor‐bearing mice, such as MGC803, Bel7402, SGC7901 mice. rhCNB induces the maturation and activation of Dendritic cells (DCs), enhances antigen presentation by APCs, promotes the phagocytic activity of macrophages and NK cells activity, and induces secretion of proinflammatory cytokines and chemokines.^9^, ^10^, ^11^ Using gene chip analysis and qPCR, we found that it up‐regulated the expression of TLRs (Toll‐like receptors) in U937 cells.^12^

Toll‐like receptors are key pattern‐recognition receptors in innate immunity that recognize pathogen‐associated molecular patterns as well as damage‐associated molecular patterns.^13^, ^14^, ^15^ Activation of TLRs triggers the secretion of cytokines and chemokines and leads to activation of innate and the adaptive immune responses.^16^, ^17^ TLRs are coming to play important roles in cancer immunotherapy.^18^, ^19^ For example, certain TLR agonists that induce immune responses have been exploited as anticancer reagent or vaccines^20^ in the clinic.

Activation of TLRs results in transcription of type 1 IFN genes and proinflammatory cytokine genes such as TNFɑ and IL‐1.^21^, ^22^ The pattern of induction of cytokines differs among the different TLRs.^23^ TLR4 was the first TLR to be identified and well‐characterized. It is the only TLR that triggers 2 parallel downstream signaling pathways.^24^, ^25^ Some exogenous or endogenous ligands of TLR4 have also been assessed for use in tumor therapy.^26^ Monophosphoryl lipid A, a derivative of lipopolysaccharide (LPS), has been used as an adjuvant with Cervarix^®^ in the prophylaxis of HPV‐associated cervical cancer. Other derivatives of LPS and TLR4 agonists such as OM174 and E6020 have also been developed.^27^, ^28^, ^29^ Some endogenous agonists of TLR4 have been tested for anticancer activity; for example, recombinant HSP70 protein has been used as a vaccine against chronic myeloid leukemia in a phase I trial.^30^, ^31^

In a previous study, we showed that rhCNB was a ligand of TLR4, and TLR4 mediated the cellular uptake of rhCNB.^32^ Since the technology for production and purification of rhCNB is simple, one can easily obtain it in abundance. Acute toxicity experiments demonstrated that a CnB dose at least 50‐fold higher than the antitumor dose was safe in mice, Successive injections of rhCNB in therapeutic doses over several months had no influence on mouse liver and spleen indexes, and safety evaluation experiments showed that CnB had almost no toxicity in vitro or in vivo (data not shown). In addition, rhCNB is very stable and does not lose antitumor activity in boiling water. Hence it may be a good anticancer candidate. However we did not establish if the antitumor and immunomodulatory actions of rhCNB were mediated by TLR4.

In this study, we confirm that the antitumor actions of rhCNB against Lewis lung cancer (LLC) cells are mediated by TLR4. We also show that rhCNB activates macrophages and promotes DC maturation in a TLR4‐dependent manner.

## 2 MATERIALS AND METHODS

### 2.1 Materials

Recombinant human CNB protein was purified in our laboratory; it was >98% pure, and endotoxin contamination was <4 EU/mg. TAK242 was from MCE (HY‐11109). C57 BL/6 wild‐type (WT) mice were from Charles River Laboratories; C57 BL/6 TLR4 knockout mice were from the Model Animal Research Centre of Nanjing University. Anti‐CD11b‐FITC, anti‐CD86‐PE, anti‐CD80‐APC, anti‐CD40‐PE, anti‐MHCII‐ PerCP‐Cy^TM^5.5, anti‐CD11c‐FITC, anti‐CD3‐FITC, anti‐CD4‐PE, anti‐CD8‐PerCP‐Cy^TM^5.5 antibodies and the isotype control antibody were from BD Biosciences. The ELISA kits for mouse TNFα, CCL5, IL‐12p40, IFN‐γ, IL4, and IL10 were from Neobioscience Technology Co., Ltd., and the mouse IFNβ ELISA kit was from Cloud Clone Corp; Recombinant mouse IL‐4 and GM‐CSF were from BioLegend.

### 2.2 Methods

#### 2.2.1 Isolation of mouse peritoneal macrophages

5 × 10^5^ mouse peritoneal macrophages from C57BL/6 WT and TLR4^−/−^ mice were plated in the wells of sterile 12‐well cell culture plates in 1 mL RPMI 1640 containing 10% FBS, and incubated overnight at 37°C with 5% CO_2_. Nonadherent cells were removed with their medium, and incubated with medium alone, or medium plus rhCNB (25, 100, 400 μg/mL).

#### 2.2.2 Quantitative RT‐PCR analysis of cytokines and chemokines

RAW264.7 cells (1 × 10^6^ cells) were incubated with 5, 25, 100 μg/mL rhCNB for 6 hours. Trizol reagent was added to each well, and RNA extracted according to standard methods. RT‐PCR was performed by the 2‐step method. Quantitative PCR was performed using SYBR Green in an Applied Biosystems 7500 Real‐Time PCR system according to the manufacturer's protocol. The sequences of the primers used were designed and synthesized by Takara.

#### 2.2.3 Isolation and induction of mouse bone marrow‐derived DCs

The bone marrow cells from C57BL/6 WT and TLR4^−/−^ mice were flushed out with RPMI 1640 medium, dispersed and passed through a 200 nylon mesh and centrifuged for 5 minutes at 1500 *g*, and erythrocytes were lysed with ACK Lysis Buffer. The remaining cells were washed twice with medium and cultured in RPMI 1640 supplemented with 10% FBS and 1% penicillin/streptomycin, in the presence of 25 ng/mL recombinant mGM‐CSF and 25 ng/mL mIL‐4. The medium was refreshed every 2 days and a portion of cells were stained with CD11c every 3 days, On day 6 of culture, nonadherent and loosely adherent cells were harvested and used in experiments as immature DCs. Samples of 1 × 10^6^ immature DCs were seeded in 12‐well plates, and the next day rhCNB was added and the cells were incubated further for 48 hours. The expression of surface molecules on the DCs was assessed by FACs.

#### 2.2.4 Flow cytometry analysis

The expression of surface molecules on RAW264.7, peritoneal macrophages, BMDCs (bone marrow‐derived cells), and T cells was analysed using a BD FACSCalibur™ flow cytometer (FCM). In brief, the cells were collected, washed and suspended in cold phosphate‐buffered saline (PBS) containing 3% (v/v) bovine serum albumin. T cells were then stained with anti‐mouse CD3, CD4, and CD8a antibodies, peritoneal macrophages were stained with CD11b, CD40, CD80, and CD86 and BMDCs were stained with CD11c CD40, CD80, CD86, and MHCII. The stained cells were washed and fixed in PBS containing 1% (v/v) bovine serum albumin and detected with a FACSCalibur™ FCM (Becton‐Dickinson, USA). Data were analyzed with CellQuest Pro software (BD Biosciences, USA).

#### 2.2.5 Determination of cytokines and chemokines

Cytokines and chemokines were measured in culture supernatants of RAW264.7, peritoneal macrophages, BMDCs, or splenocytes using commercial ELISA kits.

#### 2.2.6 Apoptosis assay

TAK242‐treated or untreated RAW264.7 cells, BMDCs and peritoneal macrophages from WT and TLR4^−/−^ mice were plated in 12‐well culture plates (2 × 10^6^ cells/well) and incubated with 100 μg/mL rhCNB for 24 hours, and culture supernatants were collected. LLC cells were plated in 6‐well plates (5 × 10^5^ cells/well) and incubated with 1:4‐diluted culture supernatants for 24 hours, and the cells were collected, and apoptosis was measured by Annexin V/PI staining; the cells were washed twice in ice‐cold PBS and stained with 10 μL Annexin V for 15 minutes in the dark at room temperature. Then 5 μL PI was added and incubation continued for 5 minutes. After staining, the cells were washed twice with PBS and analysed by FCM.

#### 2.2.7 Isolation and treatment of splenocytes

Spleens were aseptically removed from individual mice, and disrupted by scraping through nylon mesh. Erythrocytes in the spleen cell suspensions were lysed with ACK Lysis Buffer, and the viability of the splenocytes was assessed by trypan blue staining.

#### 2.2.8 The Lewis lung cancer (LLC) cells‐bearing C57BL/6 mouse model, and rhCNB administration

LLC cells suspensions (1 × 10^6^ cells) were transplanted into the right armpit of each mouse. Before inoculation, C57BL/6 WT mice were randomly divided into 3 groups of 8 mice each, receiving daily intraperitoneal injections (0.2 mL) of normal saline, rhCNB 20 mg/kg, and rhCNB 40 mg/kg, respectively. Tumor volumes were measured by Vernier calliper every 2 days from 1 week after tumor challenge. After 15 days, the tumors were removed and weighed. The mice were handled during daylight hours and the experiment was carried out in accord with the China Public Health Service Guide for the Care and Use of Laboratory Animals. Experiments involving mice and protocols were approved by the Institutional Animal Care and Use Committee of Beijing Normal University.

In another experiment C57BL/6 TLR4 knockout mice and wild‐type littermates were randomly divided into 2 groups (normal saline and rhCNB 20 mg/kg group) of 8 mice. Methods of LLC cells inoculation, rhCNB administration and tumor measurement were as above.

## 3 RESULTS

### 3.1 rhCNB induces the production of cytokines and enhances the expression of co‐stimulatory molecules by RAW264.7 macrophages in a TLR4‐dependent manner

**Figure 1:**
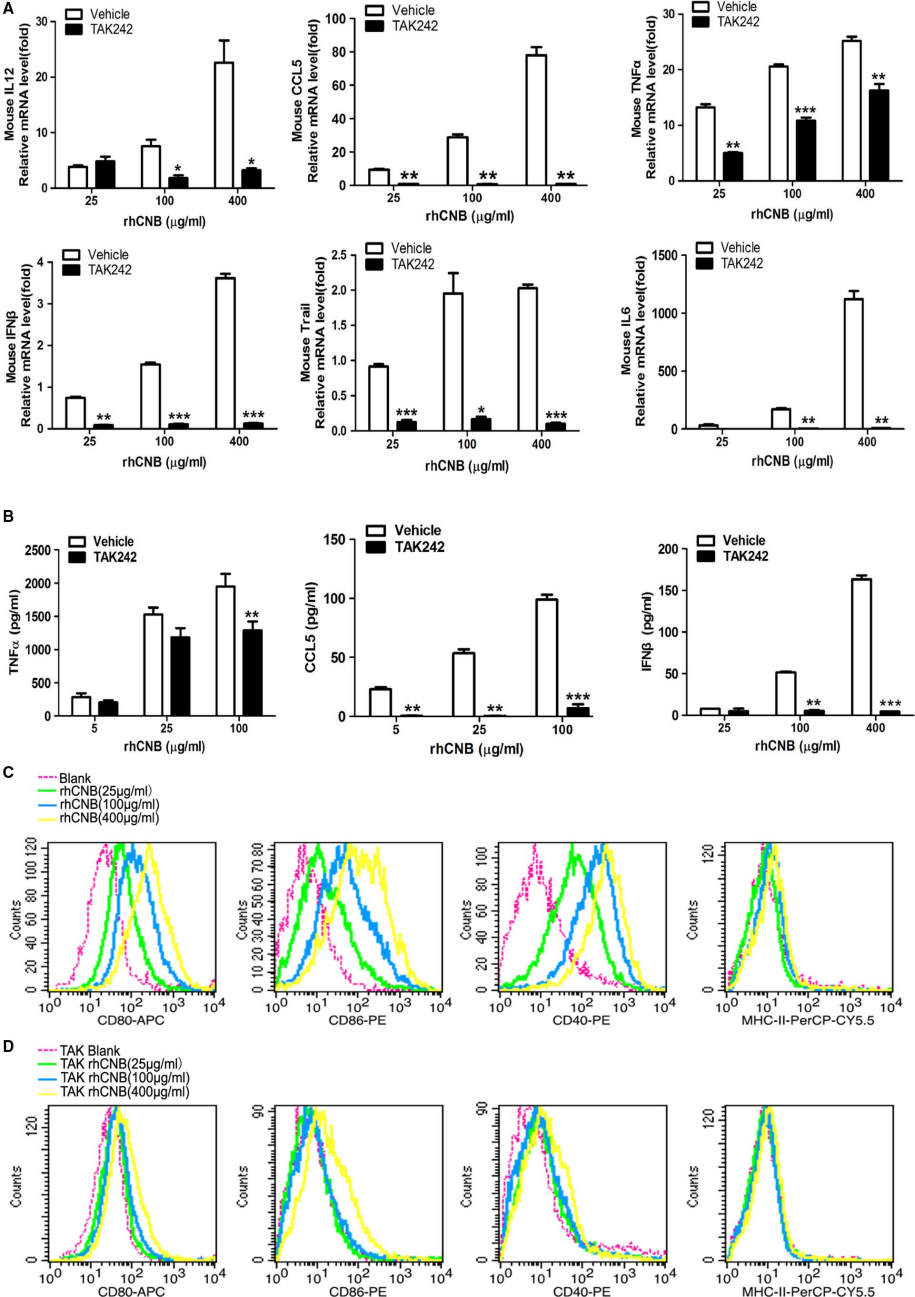
Recombinant human calcineurin B subunit (rhCNB) induces cytokine secretion and enhances the antigen‐presenting activity of RAW 264.7 macrophages via Toll‐like receptor 4 (TLR4). A, rhCNB upregulates the expression of antitumor cytokines via TLR4 in RAW264.7 macrophages. B, rhCNB triggers the secretion and production of antitumor cytokines via TLR4. C, rhCNB increases the expression of costimulatory molecules on the surfaces of RAW264.7 macrophage cell lines in a dose‐dependent manner. D, TAK242 inhibits significantly the rhCNB‐induced expression of costimulatory molecules on the surface of RAW264.7 cells. Cells were incubated with rhCNB for 24 h, and supernatants and cells were collected for ELISAs and FACs analysis. (Data are means ± SE of 3 independent experiments. *P < 0.05, **P < 0.01, ***P < 0.001)

Macrophages are key components of the innate immune system, acting to release cytokines, and present antigens to the adaptive immune system.^33^ To see whether rhCNB induces the secretion of cytokines by macrophages via TLR4, RAW264.7 cells were incubated with various concentrations of rhCNB in the presence and absence of TAK242, a specific inhibitor of TLR4^34^; cytokine transcript levels were measured by QRT‐PCR, and secreted cytokines and chemokines were quantified by ELISA. rhCNB increased transcripts of TNFɑ, IFNβ, Trail, CCL5, IL‐12 and IL‐6, and TAK242‐treatment significantly inhibited this expression (Figure 1A). Similarly, at the protein level, rhCNB induced RAW264.7 cells to produce and secrete TNF‐alpha, CCL5 and IFN‐beta, and TAK242 inhibited these processes (Figure 1B); rhCNB stimulated the production of Trail protein in a dose‐dependent manner, and TAK242 reduced this production (Figure S1).

Does rhCNB also enhance antigen‐presentation by macrophages through TLR4? We compared the expression of the costimulatory molecules CD80, CD40, and CD86 on the surfaces of TAK242‐treated and untreated RAW264.7 cells incubated with rhCNB for 24 hours. rhCNB increased the expression of these factors in the absence of TAK242 (Figure 1C), but not in its presence (Figure 1D).

### 3.2 rhCNB induces the production of cytokines and enhances the expression of costimulatory molecules of mouse peritoneal macrophages in a TLR4‐dependent manner

**Figure 2:**
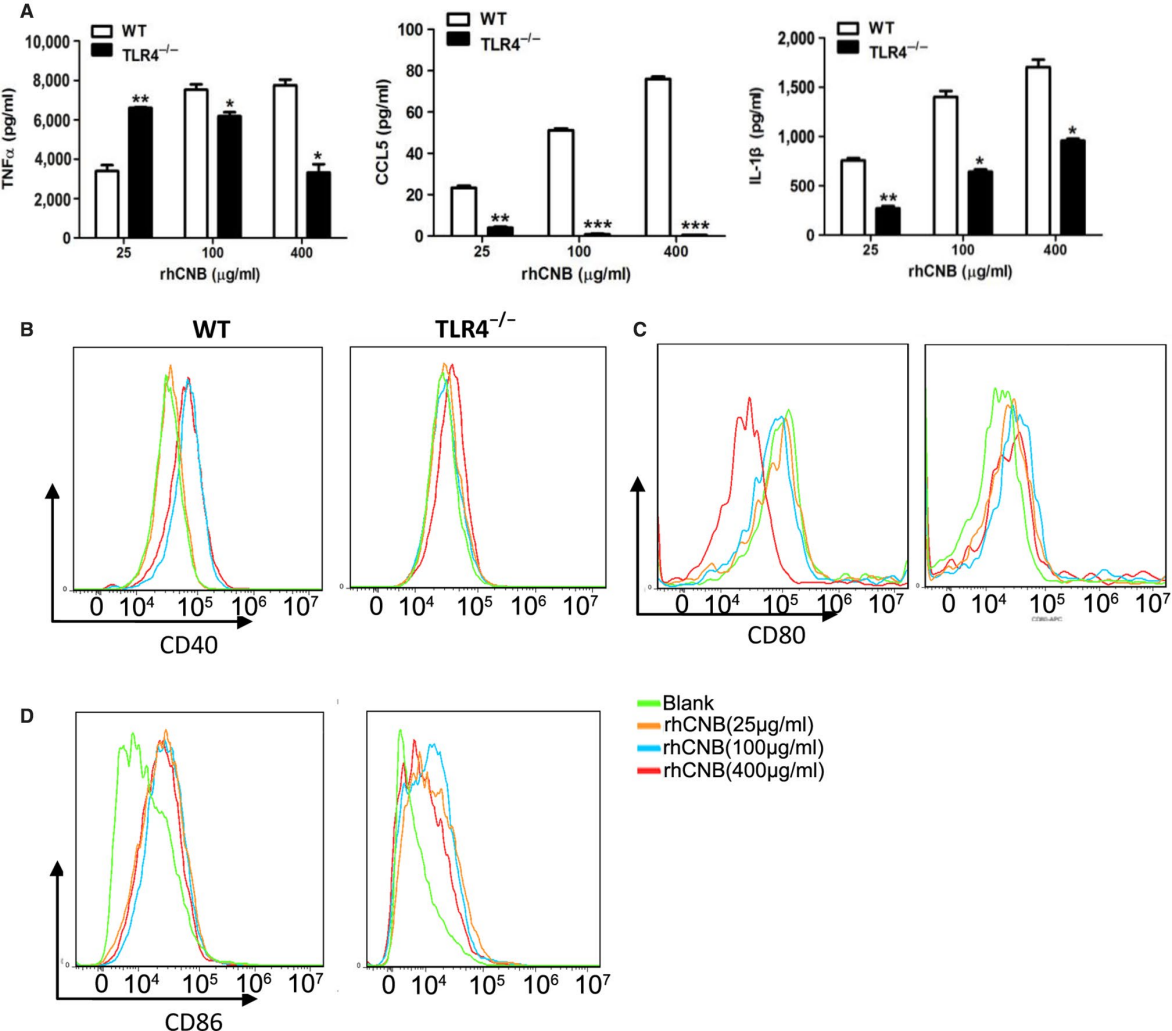
Recombinant human calcineurin B subunit (rhCNB) induces production of cytokines by mouse peritoneal macrophages in a Toll‐like receptor 4 (TLR4)‐dependent manner and enhances the expression of co‐stimulatory molecules. A, rhCNB induces cytokine secretion via TLR4 in peritoneal macrophages. B, rhCNB upregulates expression of the costimulatory molecule CD40 in peritoneal macrophages from wild‐type (WT) mice but not from TLR4^−/−^mice. C, rhCNB upregulates expression of the costimulatory molecule CD80 from peritoneal macrophages from WT mice but not from TLR4^−/−^ mice. D, The same result for CD86 expression. Peritoneal macrophages were isolated from WT and TLR4^−/−^ mice. 1 × 10^6^ cells were plated in 12‐well dishes and co‐incubated with different doses of rhCNB for 48 hours. Supernatants were collected for measuring cytokines, and cells were collected for FACs analysis. (Data are means ± SE of 3 independent experiments. *P < 0.05, **P < 0.01, ***P < 0.001)

We also analyzed the production of TNF‐ɑ, IL‐1β, and CCL5 by rhCNB‐treated peritoneal macrophages from WT mice and TLR4^−/−^ littermates. rhCNB stimulated the production of cytokines and chemokines in WT peritoneal macrophages, but not in TLR4^−/−^ peritoneal macrophages (Figure 2A). These observations indicate that the effects of rhCNB on macrophages are dependent on TLR4.

We also measured the expression of costimulatory molecules CD40, CD80 and CD86 on the surface of peritoneal macrophages in vitro. rhCNB upregulated their expression, and upregulation was dependent on the presence of TLR4 (Figure 2B‐D).

### 3.3 rhCNB activates BMDCs and up‐regulates the expression of cytokines and co‐stimulatory molecules in a TLR4‐dependent manner

**Figure 3:**
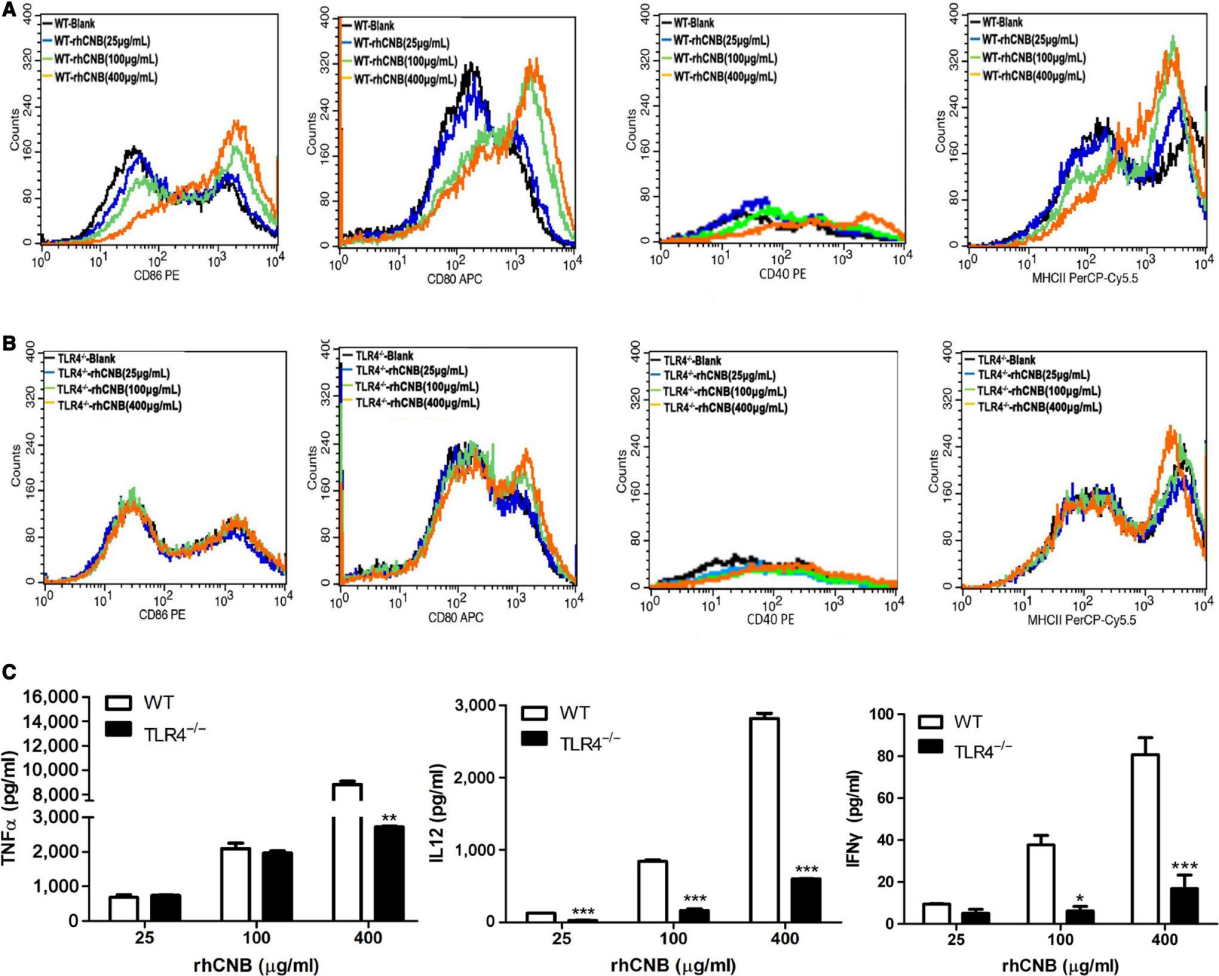
Recombinant human calcineurin B subunit (rhCNB) induces phenotypic and functional maturation of bone marrow‐derived cells (BMDCs) and cytokine secretion in a Toll‐like receptor 4 (TLR4)‐dependent manner. A, rhCNB up‐regulates the expression of costimulatory molecules and MHCII on the surface of BMDCs in wild‐type mice. B, rhCNB fails to promote expression of costimulatory molecules and MHCII on the surface of BMDCs in TLR4^−/−^ mice. C, rhCNB promotes the secretion and production of antitumor cytokines via TLR4 in BMDCs. BMDCs were treated with 25, 100, 400 μg rhCNB/mL for 48 hours, and collected for FACS analysis of CD86, 80, 40, and MHCII expression. Supernatants were also collected for assays of TNFɑ, IL‐12p40 and IFNɣ (data are means ± SEs of 3 independent experiments. *P < 0.05, **P < 0.01, ***P < 0.001)

Dendritic cells are potent antigen‐presenting cells that link innate and adaptive immunity by presenting antigens to CD4^+^ T helper cells, CD8^+^ cytotoxic T lymphocytes, and B cells. Upon activation of DCs, surface expression of MHC complexes and costimulatory molecules (CD80, CD86 and CD40, etc) is upregulated, and the corresponding cytokines are released. The activation of DCs is called maturation, and mature DCs prime T‐cell‐mediated immune responses.^35^, ^36^ To investigate the effects of rhCNB on DCs and the role of TLR4 in DC maturation, we isolated and prepared BMDCs from wild‐type and TLR4^−/−^ mice. Immature DCs were incubated with rhCNB, and levels of CD80, CD86, CD40, and MHCII were determined by flow cytometry. We found that rhCNB treatment upregulated these molecules in WT DCs (Figure 3A) but not in TLR4^−/−^DCs (Figure 3B). We also observed that rhCNB‐induced cytokine production by the DCs was dependent on TLR4 (Figure 3C).

### 3.4 The proapoptotic effect of culture supernatants of rhCNB‐stimulated immune cells on LLC cells is TLR4‐dependent

**Figure 4:**
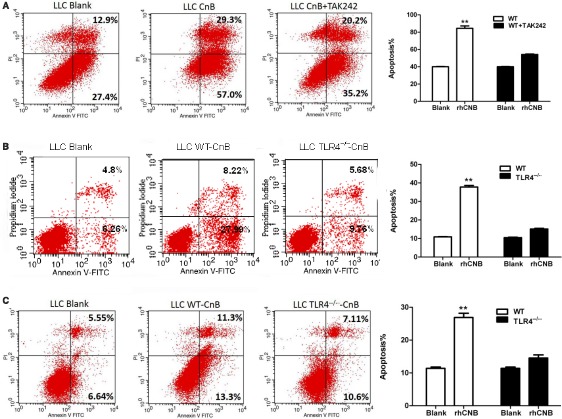
Supernatants of Recombinant human calcineurin B subunit (rhCNB)‐stimulated APCs increase apoptosis of LLC cells in a Toll‐like receptor 4 (TLR4)‐dependent manner. A, TLR4‐dependent proapoptotic effect of supernatants of rhCNB‐stimulated RAW264.7 cells on LLC cells. B, TheTLR4‐dependent proapoptotic effect of supernatants of rhCNB‐stimulated peritoneal macrophages on LLC cells. C, The proapoptotic effect of supernatants of rhCNB‐stimulated bone marrow‐derived cells on LLC cells is dependent on TLR4. LLC cells were incubated with 1:4 dilutions of 48 hours supernatants of rhCNB‐stimulated cells, and subjected to FACs analysis. (Data are means ± SE of 3 independent experiments. *P < 0.05, **P < 0.01, ***P < 0.001)

To see whether the antitumor activity of immune cells is mediated by TLR4 in vitro we collected culture supernatants of RAW264.7 cells, and rhCNB‐treated RAW264.7 cells that had been incubated in the presence and absence of TAK242. LLC cells were then incubated with dilutions of these supernatants, and apoptosis was assessed by FACS. The apoptosis rate of LLC cells exposed to supernatant of RAW264.7 cells incubated with rhCNB in the absence of TAK242 was about 86.3% (central panel of Figure 4A), compared with a rate of 55.4% (right panel of Figure 4A) using supernatant of cells incubated with rhCNB in the presence of TAK242.

We also examined the influence of supernatants of rhCNB‐treated mouse peritoneal macrophages from wild‐type and TLR4^−/−^mice on LLC cells. Supernatant from WT mouse peritoneal macrophages induced 36.2% apoptosis of LLC cells (middle panel of Figure 4B) compared with 15.4% using supernatant from TLR4^−/−^ peritoneal macrophages (right panel of Figure 4B). These data indicate that rhCNB stimulates the release of proapoptotic agents in a TLR4‐dependent manner. Similar results were obtained with culture supernatants of DCs, with apoptosis rates of 24.6% from WT DCs (middle panel of Figure 4C), and 17.7% from TLR4^−/−^DCs (right panel of Figure 4C).

### 3.5 Administration of rhCNB in vivo increases the percentages of CD4^+^ and CD8^+^ T cells in the spleen via a TLR4‐dependent process

**Figure 5:**
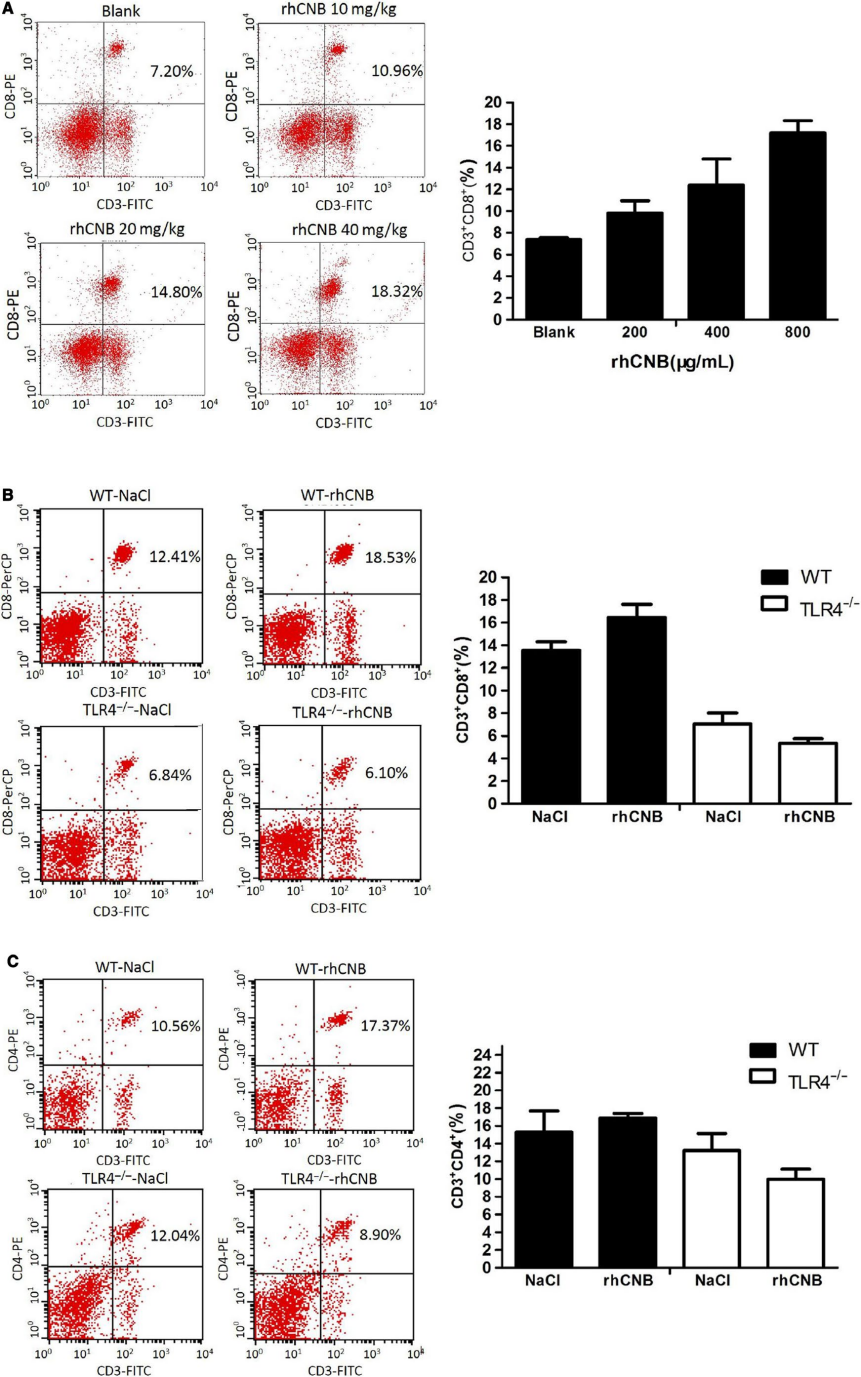
Recombinant human calcineurin B subunit (rhCNB) enhances the proportions of CD8 and CD4 T cells via a Toll‐like receptor 4 (TLR4)‐dependent process. A, rhCNB increases CD3^+^CD8^+^ proportions in splenocytes in a dose‐dependent manner B, rhCNB increases CD3^+^CD8^+^ proportions in the splenocytes of wild‐type (WT) mice, but reduces them in those of TLR4^−/−^ mice. C, rhCNB up‐regulates CD3^+^CD4^+^ proportions in the splenocytes of WT mice, but decreases them in those of TLR4^−/−^ mice. Three groups of WT mice were administered 20, 40, 80 mg/kg rhCNB, respectively, by peritoneal injection over 2 weeks. TLR4^−/−^ mice received 40 mg/kg rhCNB by peritoneal injection over 2 weeks. Splenocytes were isolated from all mice and subjected to FACs analysis

Stimulation of innate immunity leads to activation of lymphocytes and adaptive immune responses. Activation of CD4^+^ T cells and CD8^+^ CTLs (cytotoxic T cells) is important for tumor rejection.^37^ To investigate whether rhCNB provokes antitumor T cell activity in vivo, WT C57BL/6 mice were injected intraperitoneally with different doses of rhCNB (10, 20, 40 mg/kg) or NaCl (as control) for 2 weeks, and splenocytes were isolated and harvested to detect CTLs by FACs. As shown in Figure 5A, the proportion of CD3^+^CD8^+^ cells increased in a manner dependent on rhCNB dose. To examine the role of TLR4 in this T cell increase, we analysed the splenocytes of TLR4^−/−^ mice injected with rhCNB. We found that rhCNB treatment did not increase the proportion of CD3^+^CD8^+^ CTL cells in the splenocytes of the TLR4^−/−^ mice, but rather reduced it slightly (Figure 5B), and the same was true for the proportions of CD3^+^CD4^+^ cells (Figure 5C).

### 3.6 rhCNB inhibits tumor growth in LLC tumor‐bearing mice, and the tumoricidal activity is mediated by TLR4

#### 3.6.1 rhCNB inhibits tumor growth in LLC tumor‐bearing mice

**Figure 6:**
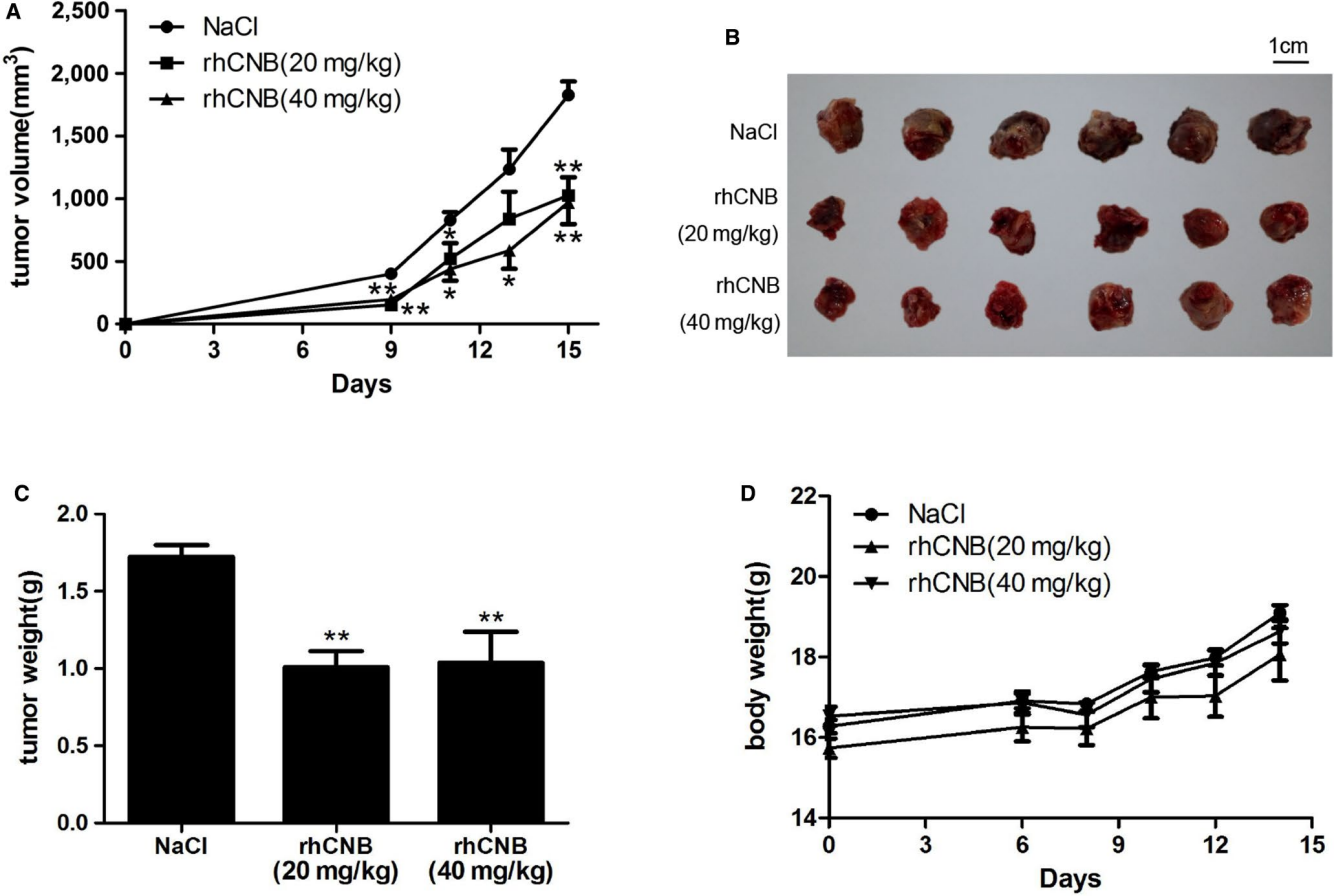
Antitumor activity of Recombinant human calcineurin B subunit (rhCNB) against LLC murine tumor cells. LLC cells were inoculated into the wild‐type mice, and the mice received daily intraperitoneal injections (0.2 mL) of normal saline, rhCNB 20 mg/kg, and rhCNB 40 mg/kg, respectively. Tumor volumes were measured every 2 days. After 15 days, the tumors were removed and weighed. A, B and C. rhCNB reduced LLC tumor volumes, size and weight, and inhibited significantly LLC tumor growth compared the control group. D. The body weights of mice did not change obviously. (n = 6, data are means ± SE. *P < 0.05, **P < 0.01, ***P < 0.001 compared with the control NaCl group)

In order to assess the antitumor activity of rhCNB, LLC cells suspensions (1 × 10^6^ cells) were transplanted into the right armpits of C57BL/6 WT. Before inoculation, the mice were randomly divided into 3 groups of 8 mice each, and received daily intraperitoneal injections (0.2 mL) of normal saline, rhCNB 20 mg/kg, and rhCNB 40 mg/kg, respectively, as described in Methods. After 2 weeks of treatment, the tumor volumes in the rhCNB‐treated groups were lower than in the NaCl control group (Figure 6A). After the last injection, the tumors were isolated and weighed, and the weights of the rhCNB‐treated groups were significantly lower than those in the control group; tumor growth was inhibited by about 50%, but the antitumor effect in the group receiving injections of 40 mg/kg of rhCNB was no greater than in the group receiving 20 mg/kg of rhCNB (Figure 6B,C). The body weights of the rhCNB‐treated groups were not obviously different from those of the control group (Figure 6D). These results indicate that rhCNB is effective in inhibiting LLC tumor growth.

#### 3.6.2 The antitumor effects of rhCNB on LLC tumors are mediated by TLR4

**Figure 7:**
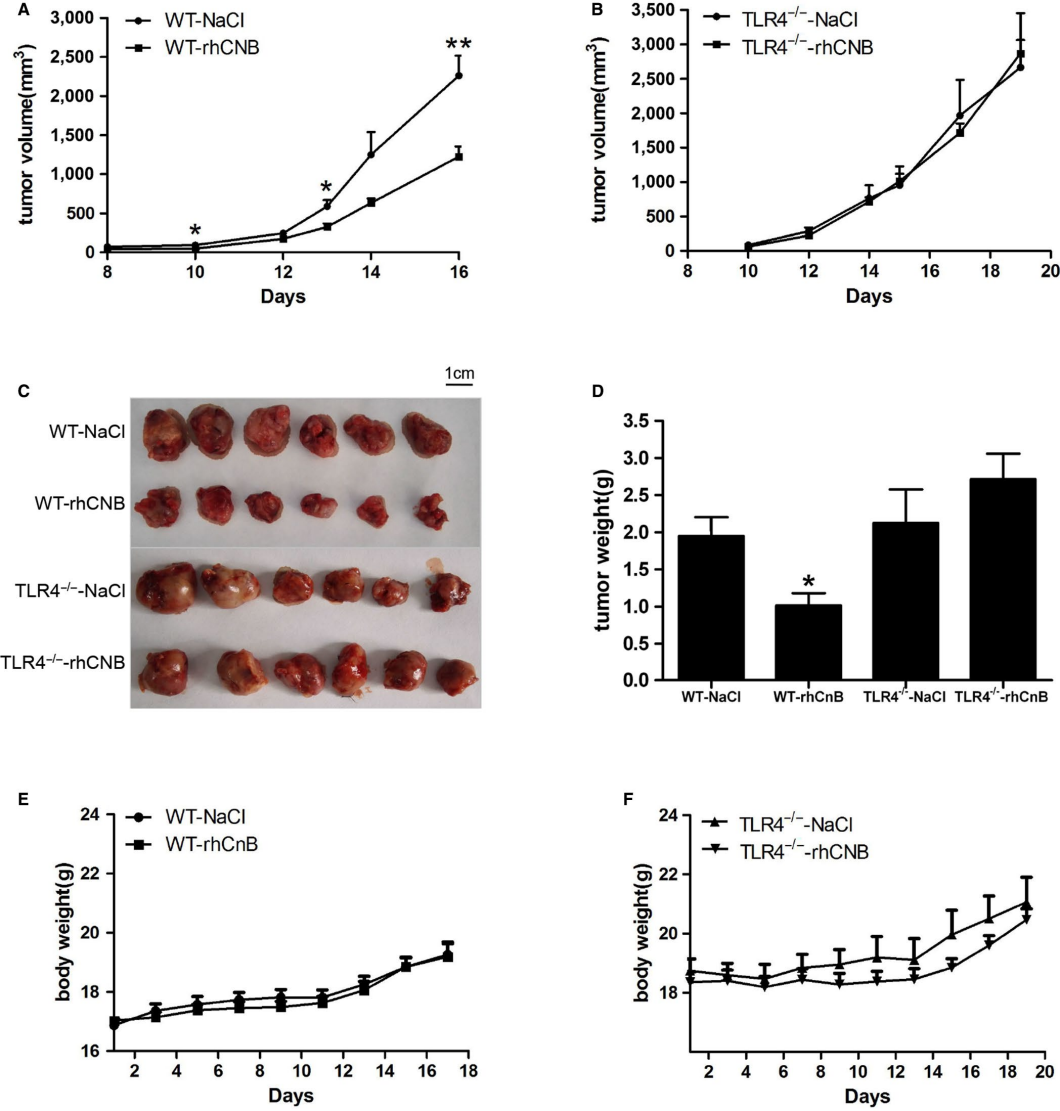
The antitumor effects of Recombinant human calcineurin B subunit (rhCNB) on LLC tumors are mediated by Toll‐like receptor 4 (TLR4). LLC cells were inoculated into the TLR4^−/−^ mice and their wild‐type littermates, and the mice received daily intraperitoneal injections (0.2 mL) of normal saline and rhCNB 20 mg/kg, respectively. Tumor volumes were measured every 2 days. After 15 days, the tumors were removed and weighed. A, B, C, and D, rhCNB inhibits tumor growth in LLC‐bearing wild‐type mice, but fails to inhibit tumor growth in LLC‐bearing TLR4^−/−^ mice. E, The administration of rhCNB did not influence the body weights in the LLC‐bearing wild‐type mice. F, The administration of rhCNB increased slightly the body weights in the LLC‐bearing TLR4^−/−^ mice. (Data are means ± SE. n = 6, *P < 0.05, **P < 0.01, ***P < 0.001 compared with the control group)

To assess whether the antitumor effects of rhCNB is mediated by TLR4 in vivo, LLC cells suspensions (1 × 10^6^ cells) were transplanted into the right armpits of C57BL/6 TLR4 knockout mice and their littermates, and they were given daily intraperitoneal injections (0.2 mL) of normal saline or rhCNB 20 mg/kg, again rhCNB caused a progressive reduction in tumor size in the wild‐type mice with increasing length of administration (Figure 7A). However rhCNB did not cause any obvious inhibition of tumor growth in the TLR4^−/−^mice, (Figure 7B). Similarly rhCNB reduced tumor weight in the wild‐type mice but not in their TLR4^−/−^ littermates (Figure 7C,D). There was no obvious change in the body weight of the rhCNB‐treated wild‐type mice compared with the control group (Figure 7E), whereas the body weight of the rhCNB‐treated TLR4^−/−^group increased slightly (Figure 7F). These results demonstrate that the antitumor activity of rhCNB is mediated by TLR4.

## 4 DISCUSSION

We have shown previously that rhCNB is a good candidate for use in tumor therapy. We demonstrated that rhCNB is an immune‐stimulatory protein, and exerts antitumor effects by activating cells of the innate immune system, but the molecular target of rhCNB remained unclear. To accelerate the development of rhCNB as an antitumor treatment, it is very important to further understand the mechanism of its antitumor action. In this study, we showed that it is an agonist of TLR4 and that its tumoricidal activity is mediated by TLR4. Activation of TLR4 induces DCs and other antigen‐presenting cells to secrete cytokines and promote DC maturation and antigen uptake and presentation. This leads in turn to activation of downstream T cell immunity in the form of differentiated CD4^+^ T cells (Th1, Th2, and Th17), and CD8^+^ CTL expansion.^38^, ^39^ We showed also that rhCNB induces the production and secretion of cytokines TNFɑ, IL‐1β, and IL‐12p40 as well as chemokine CCL5, upregulates the expression of MHCII and costimulatory molecules (CD80, CD86, and CD40) on the surface of DCs and macrophages, and promotes DCs maturation. Since the activation of DCs and macrophages leads to the expansion of CD4^+^ and CD8^+^ T cells,^40^ rhCNB is effective in stimulating antitumor immunity.

Previously we showed that rhCNB induced the regression of tumors derived from subcutaneously implanted liver cancer and gastric cancer cells. In the present work, we implanted malignant LLC cells into WT mice. The administration of rhCNB reduced significantly the growth of these cells, showing that rhCNB is also effective against lung cancer cells. In the study, we also identified the immediate target of rhCNB antitumor activity. WT and TLR4^−/−^mice were administered rhCNB for 2 weeks. The rhCNB treatment significantly inhibited LLC growth in the WT mice but not in the TLR4^−/−^ mice. Therefore, our findings establish that the antitumor effects of rhCNB are mediated by TLR4.

Culture supernatants of rhCNB‐stimulated APCs caused significant LLC apoptosis in vitro, primarily because rhCNB induced the production and secretion of antitumor cytokines such as Trail, TNFɑ, IFNβ, IFNγ etc We found that 400 μg/mL rhCNB induced RAW264.7, DCs and macrophages to produce about 8 ng TNFɑ, which is a tumor necrosis factor and can trigger apoptosis, and perhaps the apoptosis induced by culture supernatants of rhCNB‐stimulated APCs is primarily triggered by TNFɑ. In additon, production of TNFɑ was significantly inhibited at higher rhCNB concentrations but TNFɑ production at lower concentration of rhCNB was similar in TLR4‐deficient and WT cells. Since it appeared that rhCNB induced TNFɑ production via some component in addition to TLR4, we examined the production of TNFɑ and CCL5 in wild‐type and TLR2‐deficient cells and observed that the production of TNFɑ in the TLR2‐deficient cells was significantly inhibited at higher rhCNB concentrations but not at lower concentrations while the production of CCL5 was not obviously affected (data not shown), and the results indicated TNFɑ production via the 2 receptors of TLR2 and TLR4 at higher concentration of rhCNB.

The average tumor weight of the rhCNB‐treated TLR4^−/−^mice was a little higher than that of the NaCl control mice. There is increasing evidence that TLR4 is not only expressed on immune cells, but also on a large number of tumor types.^41^, ^42^ Because TLR4 expression by tumor cells may promote their own proliferation, survival, or even immunosuppression, activation of TLR4 is seen as a “double‐edged sword” in terms of therapeutic effects.^43^ Since specific antitumor immune responses are beneficial for tumor therapy, ways to specifically harness the antitumor effects of TLR4 stimulation need to be further explored.

In previous research we reported that rhCNB is a ligand of CD11b,^44^ an essential component of the TLR4 complex,^45^ and that it induced Trail expression in RAW264.7 macrophages.^46^ In this study, we confirmed that rhCNB induced Trail expression in RAW264.7 macrophages and that reducing TLR4 expression significantly downregulated the expression of Trail.

In summary, we have clarified the mechanism of the antitumor and immunomodulatory actions of rhCNB, and have shown that it acts via the TLR4. There is increasing evidence that TLR agonists can act as anticancer agents,^20^, ^31^, ^47^ and rhCNB appears to hold promise as such an anticancer agent.

## CONFLICT OF INTEREST

The authors declare no conflict of interests.
