# Supplementary material for: The immunostimulatory effects and pro‐apoptotic activity of rhCNB against Lewis lung cancer is mediated by Toll‐like receptor 4
Source: Cancer Med. 2019 Jun 20;8(9):4441–53. doi: 10.1002/cam4.2158 (PMC6675711; doi:10.1002/cam4.2158)
Supplement: Supplementary file 1 [file CAM4-8-4441-s001.pdf]

The immunostimulatory effects and pro-apoptosis activity of rhCNB against LLC is mediated by Toll-like receptor 4.

Jinju Yang[1, 2], Hongwei Zhang[1], Ziwei Zhu, Rui Yang[1], Yadan Gao[1], Benqiong Xiang[1]* and Qun Wei[1]*

[1]Department of Biochemistry and Molecular Biology, Beijing Normal University, Gene Engineering and Biotechnology Beijing Key Laboratory, Beijing, 100875, P. R. of China; [2]National Key Laboratory of Biochemical Engineering, Institute of Process Engineering, Chinese Academy of Sciences, Beijing 100190, China.

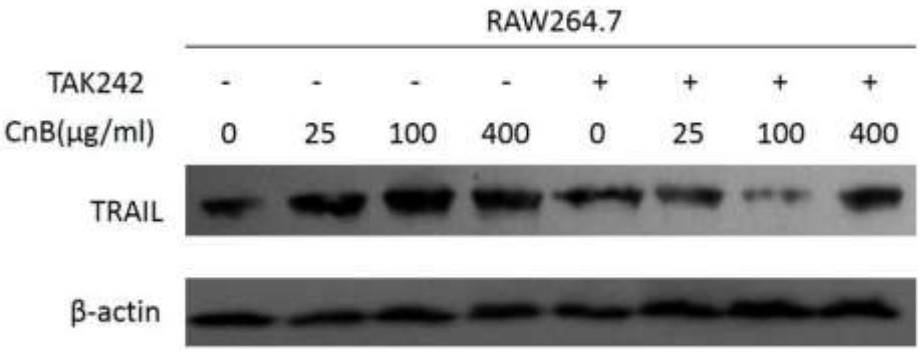

Figure S1. rhCNB up-regulated the expression of the trail protein in a dose-dependent manner, but TAK242 inhibited the expression. 1x10^6 RAW264.7 cells were plated in 12- well dishes and co-incubated with the different doses of rhCNB in the absence or presence of TAK242 for 48h. the cells were collected for western blot analysis.
